# Temporal blood flow changes measured by diffuse correlation tomography predict murine femoral graft healing

**DOI:** 10.1371/journal.pone.0197031

**Published:** 2018-05-29

**Authors:** Songfeng Han, Ashley R. Proctor, Jingxuan Ren, Danielle S. W. Benoit, Regine Choe

**Affiliations:** 1 Institute of Optics, University of Rochester, Rochester, NY, United States of America; 2 Department of Biomedical Engineering, University of Rochester, Rochester, NY, United States of America; 3 Department of Orthopaedics and Center for Musculoskeletal Research, University of Rochester Medical Center, Rochester, NY, United States of America; 4 Department of Chemical Engineering, University of Rochester, Rochester, NY, United States of America; 5 Department of Biomedical Genetics, University of Rochester Medical Center, Rochester, NY, United States of America; 6 Department of Electrical and Computer Engineering, University of Rochester, Rochester, NY, United States of America; Louisiana State University, UNITED STATES

## Abstract

Blood flow changes during bone graft healing have the potential to provide important information about graft success, as the nutrients, oxygen, circulating cells and growth factors essential for integration are delivered by blood. However, longitudinal monitoring of blood flow changes during graft healing has been a challenge due to limitations in current techniques. To this end, non-invasive diffuse correlation tomography (DCT) was investigated to enable longitudinal monitoring of three-dimensional blood flow changes in deep tissue. Specific to this study, longitudinal blood flow changes were utilized to predict healing outcomes of common interventions for massive bone defects using a common mouse femoral defect model. Weekly blood flow changes were non-invasively measured using a diffuse correlation tomography system for 9 weeks in three types of grafts: autografts (N = 7), allografts (N = 6) and tissue-engineered allografts (N = 6). Healing outcomes were quantified using an established torsion testing method 9 weeks after transplantation. Analysis of the spatial and temporal blood flow reveals that major differences among the three groups were captured in weeks 1–5 after graft transplantation. A multivariate model to predict maximum torque by relative blood flow changes over 5 weeks after graft transplantation was built using partial least squares regression. The results reveal lower bone strength correlates with greater cumulative blood flow over an extended period of time (i.e., 1–5 weeks). The current research demonstrates that DCT-measured blood flow changes after graft transplantation can be utilized to predict long-term healing outcomes in a mouse femoral graft model.

## Introduction

More than 2.2 million graft procedures are performed in the clinic every year to treat critical-sized bone defects [1], which will not heal without intervention [2]. The “gold-standard” allograft treatment uses processed bone material from a cadaver and is advantageous in terms of the amount of available bone material. However, allografts have a 60% failure rate over 10 years post-implantation [1, 3, 4]. To avoid infection or immunological responses in patients, allografts must be thoroughly cleaned to remove all living tissue. Poor healing typical of allografts is due to loss of the periosteum: a thin layer of tissue covering the bone [3, 5]. On the other hand, autograft procedures that utilize healthy autologous bone tissue with periosteum from a non-load-bearing skeletal region usually achieve complete healing, but this approach is limited by lack of available tissue volume and donor site morbidity.

To improve allograft healing, tissue engineered (T.E.) periosteum-mimetic approaches have been proposed and tested in preclinical studies using a murine femoral graft model [6, 7]. To enable comparison of healing outcomes among different grafts, various physiological parameters were measured, including bone callus volume quantified from high-resolution micro-Computed Tomography (micro-CT) on isolated femurs, microvessel density calculated from lead-contrast micro-CT, and bone strength quantified by biomechanical testing [8, 9]. These quantification methods are end-point and require mouse sacrifice. As a result, a large number of mice are required to track group-averaged longitudinal changes during healing.

Noninvasively tracking individual longitudinal changes and predicting the efficacy of new tissue engineering methods remain a challenge using current imaging techniques. Diffuse correlation tomography (DCT) is an emerging technique that can non-invasively monitor 3-dimensional blood flow distribution [10, 11]. It uses a near-infrared laser as the coherent light source. Measurement is non-invasive with minimal cost [12, 13]. DCT has been studied in both preclinical and clinical settings to monitor blood flow changes in mouse and rat brains [14, 15], and in human breast [16]. We have developed a non-invasive DCT system to monitor 3-dimensional blood flow changes in the mouse femur [17, 18]. With the DCT system, we have revealed that blood flow changes are different among three groups of autografts, allografts, and T.E. allografts. In this paper, we directly utilize blood flow changes monitored by DCT to predict the long-term healing outcomes, with the ultimate goal to establish DCT as a non-invasive tool to quantitatively assess graft healing.

Weekly DCT measurements were performed on three groups of mice for 9 weeks, adhering to the same protocol reported previously [17]. To independently quantify graft healing, we utilized an established biomechanical testing method to measure maximum bone torque after 9-week healing, which has been widely used as an indicator for long-term healing [6, 8, 19]. We investigated the spatial and temporal features in blood flow changes to select the most relevant data; then used partial least squares (PLS) regression to build a multivariate model to predict maximum torque using blood flow changes from week 1 to 5.

## Methods

### Murine femoral graft model

This study was approved by the University Committee on Animal Resources (UCAR) at the University of Rochester (Permit number: UCAR-2010-056). 5-week-old female BALB/c mice were purchased from the Jackson Laboratory (Bar Harbor, ME) and housed according to the UCAR mouse cage density policy (<5 mice per cage) in an animal facility with a 12:12 light-dark cycle. Water and food was available to mice *ad libitum*.

#### Preparation of hydrogel-based tissue engineered periosteum

**Hydrolytically degradable hydrogel precursors** - Poly(ethylene glycol)-based hydrolytically-degradable tri-block copolymers (methacrylate-poly(lactide)-b-PEG-b-poly(lactide)-methacrylate) were synthesized via a ring-opening polymerization of *d*,*l*-lactide onto PEG (Alfa Aesar, MW 10 kDa) followed by microwave-assisted methacrylation, as previously described [8, 20–22]. ^1^H-NMR analysis (Bruker Avance 400 MHz, CDCl_3_) was used to determine the presence of 3 lactide units and >95% methacrylate functionalization per PEG macromer (-*CH2CH2O*- (PEG), 908H, 3.2–3.8 ppm, multiplet; -*OCH*(CH3)COO-, 4H/PLA repeat, 5.2–5.3 ppm, multiplet; -OCH(*CH3*)COO-, 12H/PLA repeat, 1.4–1.6 ppm, multiplet; CH2 = C(CH3)-, 4H/macromer, 5.6 and 6.3 ppm, singlets; CH2 = C(*CH3*)-, 6H/macromer, 1.9 ppm, singlet).

The cell adhesive sequence Arg-Gly-Asp-Ser (RGDS; 433 Da, EMD Chemicals, San Diego CA) was coupled to acrylate-PEG-N-Hydroxysuccinimide (Jenkem Technology, MW 3500 Da) through the amino terminus to allow for incorporation into hydrogels, as previously described [8, 21]. The product (Acrylate-PEG-RGDS) was dialyzed against deionized water (molecular weight cutoff = 1000 Da, Spectrum Labs), lyophilized, analyzed via matrix-assisted laser desorption/ionization-time of flight mass spectrometry (MALDI-TOF, Bruker AutoFlex III SmartBeam); (solvent: 50% acetonitrile in H2O + 0.1% TFA; matrix: α-cyano-4-hydroxy cinnamic acid (TCI Europe); calibrant: Peptide Calibration Standard (Bruker, #206195)) (m/z Cl-, 3854 Da), and stored at 4°C.

**Mouse mesenchymal stem cells** - Bone marrow derived mouse mesenchymal stem cells (mMSCs) isolated from Balb/c mice were obtained from the mesenchymal stem cell distribution center at Scripps Research Institute (Jupiter, Florida). mMSCs were cultured at 37°C, 5% CO_2_, and hypoxic conditions (5% O_2_), afforded by a BioSpherix control system (BioSpherix Ltd.) in α-minimum essential medium with L-glutamine (Gibco) supplemented with 10% fetal bovine serum (Atlanta Biologicals), 100 units/ml penicillin, 100 μg/ml streptomycin, and 0.25 μg/ml amphotericin B. mMSCs were used prior to passage 5.

**Tissue engineered periosteum formation** - As previously described [7, 8, 19], a 10 wt% solution of PEG-PLA-DM and 2.0 mM Acrylate-PEG-RGDS in PBS was combined with the photoinitiator lithium phenyl-2,4,6-trimethylbenzoylphosphinate (LAP, synthesized as previously described [23]), at a final concentration of 0.05 wt%. Trypsinized mMSCs were added to the PEG macromer solution to achieve a final concentration of 25 million cells/mL. 20 μL of the PEG/cell solution was pipetted into custom-made cylindrical molds containing allografts which were subsequently exposed to long-wavelength 365 nm light (5 mW/cm^2^) for 10 min at room temperature to form uniform PEG/cell hydrogel coatings with >95% cell viability [24, 25].

#### Graft surgery

To create the femoral graft, first, a 6-week-old BALB/c mouse was anesthetized with isoflurane, and a bone defect was created by removing a 4-mm mid-diaphyseal segment of the left femur using a Dremel with an attached double-sided diamond disc (Brasseler USA, Savannah, Georgia). Then, a 4-mm graft was transplanted into the defect and stabilized with the remaining host femur using a 26G intramedullary pin. Three graft groups were created. The autograft group received the removed femur segment from itself with its orientation flipped. The allograft group received a devitalized femur segment from a genetically different strain of mice (i.e., C57BL/6). The devitalized femur segment was prepared by removing the periosteum, flushing the medullary canal with phosphate buffered saline (PBS), sterilizing the bone with 70% ethanol, and rinsing the bone with PBS prior to storage at -80°C for 1 week before transplantation. The T.E. allograft group received the same kind of processed bone material as the allograft group except that the femur segment was coated with the hydrogel-based T.E. periosteum described in the previous section. After the surgery, analgesic drugs were administrated every 12 hours for 3 days or until no sign of pain was observed in the mouse, whichever was longer.

Initially, 7 mice were allocated for each group. The sample size was determined so that there were enough samples to repeat the variability in temporal blood flow change observed previously [17], and extra subjects in each group to account for potential attrition in the experiment. Healthy 6-week-old BALB/c mouse mice were randomly allocated to each group. Each type of graft transplantation was performed on the same day to minimize the variation within the same group. However, one mouse in the allograft group died two days after surgery due to conspecific aggression and one mouse in the T.E. allograft group was excluded because of insufficient mMSCs during T.E. allograft preparation. As a result, DCT measurements were performed on 19 mice in total, with 7 (Mouse 1 to 7) in the autograft group, 6 (Mouse 8 to 13) in the allograft group and 6 (Mouse 14 to 19) in the T.E. allograft group.

### Diffuse correlation tomography

**Fig 1:**
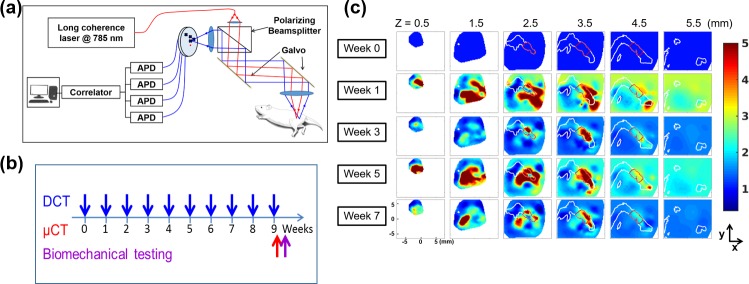
DCT measurements and results. (a) DCT optical measurement system set-up (modified from figure in ref [17]); (b) The timeline of measurements for each mouse; (c) An example of the three-dimensional relative blood flow (*rBF*) changes in a mouse with an allograft (Mouse 10). Each row shows the z-slice of the three-dimensional *rBF* distribution at each week and each column shows the temporal changes for a specific z-slice. The borders of the bones and graft are outlined with white and red lines, respectively. Note that weekly DCT measurements were made, but only results from selected weeks are shown here.

Longitudinal DCT measurements were performed one day before graft transplantation (week 0), and then every week after the surgery for 9 weeks. DCT measurements were performed using the same diffuse correlation tomography system and source-detector configuration as described previously [17]. The measurement set-up is illustrated in Fig 1A. Briefly, a near-infrared laser source coupled with multimode fibers was used to illuminate the tissue, and single-mode fibers were utilized to collect multiply-scattered light. The source and detector fibers were imaged on the tissue surface using two optical lenses. By changing the angle of two galvanized mirrors inserted between the lenses, the source-detector pattern was scanned on the tissue surface in two dimensions. The source formed a 0.2 mm spot on the surface with an input power around 1 mW. Multiply-scattered light collected at 3.0, 4.5, 6.0 and 7.5 mm away from the source was fed to 4 avalanche photodiodes (APDs). A 4-channel correlator calculated the intensity temporal autocorrelation function of the detected light signal every 2 seconds and the results were stored in a computer. The mouse was anesthetized with isoflurane for each 15-minute DCT scan. After the last DCT measurement at week 9, a micro-CT scan was performed on the mouse leg with a spatial resolution of 35 μm. A finite element mesh of the mouse geometry was created from the micro-CT yielded anatomical structure using NIRFASTSlicer, a free software for medical image segmentation [26]. The data acquisition timeline is illustrated in Fig 1B.

With the measured data, three-dimensional blood flow distribution was reconstructed with an iterative reconstruction method [17], yielding blood flow index *αD*_*b*_(**r**,*t*;*i*), where *α* represents the fraction of photon scattering events occurring from moving red blood cells with respect to static scatterers, *D*_*b*_ is the particle diffusion coefficient, **r** is the spatial position, *t* is the measurement week number, ranging from 0 to 9, and *i* is the number of the mouse (1 to 19 for this experiment). In particular, the micro-CT based finite element mesh was utilized to assign different optical properties for the soft tissues and the bone in order to improve the fidelity of *αD*_*b*_. More details of the image reconstruction are available in Reference [17]. Relative blood flow (*rBF*) change was calculated by normalizing the blood flow distribution of different weeks by the distribution at the pre-surgical time point, week 0,

$$rBF\left(r,t;i\right)=\frac{\alpha D_{b}\left(r,t;i\right)}{\alpha D_{b}\left(r,0;i\right)}.$$ (1)

As an example, three-dimension longitudinal blood flow changes that occur within an allograft during the healing process are shown in Fig 1C, with each row showing the three-dimensional distribution at each time point and each column showing the temporal trends at each depth z from the tissue surface. The borders of the bone are outlined with white lines, and those of the graft are outlined with red lines.

### Biomechanical testing

To quantify the healing of grafted femurs, biomechanical testing was performed after the last DCT measurement at week 9 after transplantation [9]. Studies have shown that biomechanical testing at week 9 reveals significant differences among the three graft types [8, 19]. To prepare for testing, mice were sacrificed after micro-CT, and the femur with the graft was harvested and stored at -80°C. On the day of biomechanical testing, the femur was thawed and the intramedullary pin was removed. Femurs were cemented in aluminum cubes on both ends using poly(methyl methacrylate), with a 6-mm gap at the center exposed. Then, the aluminum cubes were mounted in the two arms of a rotation platform (200 N-mm torque cell; Bose Corp., Minnetonka, MN). After starting the testing, the two arms of the testing platform were rotated in opposite directions at a constant speed to exert torsion on the femur until failure. The strain-stress curve was recorded during testing and utilized to derive maximum torque. Nineteen grafted femurs were harvested; however, tests on seven femurs were not possible due to graft damage upon intramedullary pin removal. As a result, the biomechanical testing was successfully performed on 12 mice, with 4 mice in each group.

### Spatiotemporal blood flow change analysis

To build a predictive model for the healing outcomes based on *rBF*, the first challenge was to account for the disproportional size of the spatiotemporal *rBF* information (~ tens of thousands of spatial positions X 9 independent time points for each mouse) compared to maximum torque (one value for each mouse). The large size of the data would easily result in overfitting the model. To mitigate this problem, the following analyses were performed to reduce data dimensions.

**Fig 2:**
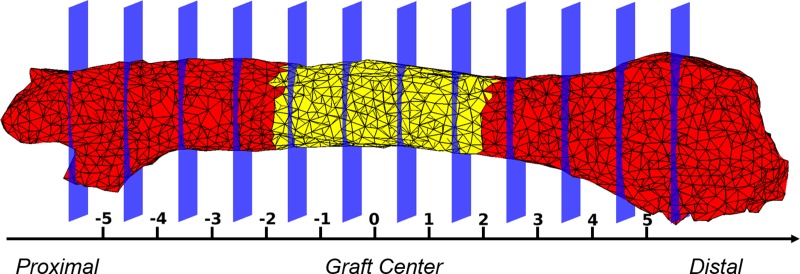
Segmentation of a femur into 1 mm segments to calculate the average of relative blood flow (*rBF*). Each femur segment was represented by its distance from the center of the graft, with negative values representing segments closer to the proximal end (connecting to the pelvis) and positive values closer to the distal end (connecting to the tibia). The yellow part in the center denotes the inserted graft, and the red parts are the parts of the host femur.

First, average blood flow changes were calculated in each 1 mm segment of the femur. To segment the femur: first, the nodes of the femur were extracted from the finite element mesh using k-means clustering [27]. Second, a femur axis was defined and used as a reference to segment the femur. To get the axis, principle component analysis (PCA) was performed on the nodes of the femur. The first principal component from PCA was utilized as the femur axis. Third, the center of the inserted graft region was calculated and set as the origin of the femur axis. Finally, the femur was divided into 1 mm segments along the axis. The segmentation algorithm was implemented using MATLAB (MathWorks, Natick, MA). An example of the femur segmentation is shown in Fig 2. Each femur segment was represented by its distance relative to the center, with negative values representing segments closer to the proximal end (connecting to the pelvis) and positive values closer to the distal end (connecting to the tibia).

With segmentation, the average *rBF* for each femur segment at different weeks was calculated, yielding *rBF*(*l*,*t*;*i*), where *l* represents the position of the segment along the femur, *t* represents the time for DCT measurement, and *i* represents the mouse number (Supporting Information S1 Table). For each mouse, spatiotemporal *rBF* change can be visualized by plotting *rBF*(*l*,*t*;*i*) as a surface in the z axis with week number *t* and the segment position *l* as the x and y axis, respectively. To visualize the changes, the average spatiotemporal *rBF* over all mice were examined.

Average *rBF* in the graft region, *rBF*^g^, was calculated as it shows the major temporal trend in blood flow change. *rBF*^g^(*t*;*i*) was calculated as the average of *rBF* between *l* equals -2 to 2 for mouse *i* at week *t*. *rBF*^g^ for each mouse was examined as well as the group average. Shapiro-Wilk was used to test the normality of *rBF*^g^(*t;i*) distribution among the mice at different time *t*. To alleviate the skewness in the distribution, logarithm transformation was performed to obtain log(*rBF*^g^). Then, Pearson’s correlation coefficients were calculated between log(*rBF*^g^) of different weeks, forming the correlation matrix. *p* < 0.05 was deemed statistically significant.

### Partial least squares regression model

To predict healing outcomes (i.e., responses) using blood flow measurements, partial least squares (PLS) regression was utilized to build a multivariate linear model using blood flow results from weeks 1–5 (i.e., predictors). PLS regression attempts to fit new factors (i.e., linear combinations of the predictors or responses) that maximize the covariance between original predictors and responses [28], thus reducing the dimensionality of the regression model. It has robust performance in situations where there are few samples and when the predictor variables are highly correlated. PLS has been widely used in analytical chemistry and Raman spectroscopy [29, 30]. In our analysis, the PLS method was implemented using the statistical software JMP (SAS Institute, Cary, NC) with Nonlinear Iterative Partial Least Squares (NIPALS) algorithm and k-fold cross-validation method. Based on data from 12 mice with the available maximum torque measurements, the PLS regression algorithm model generated the multivariate model with minimal root mean Prediction Residue Sum of Squares (PRESS) as well as Variable Importance in the Projection (VIP). PRESS quantifies the difference between the actual results and predicted results in the cross-validation. A model with a lower PRESS performs better than the one with a higher PRESS. VIP values quantify the importance of each predictor in forming the multivariate model. Predictors with a VIP value greater than 0.8 are usually considered important by JMP software [28, 31]. Data used in the different analyses are summarized in the Supporting Information S1 Fig.

## Results

### Biomechanical testing results

**Fig 3:**
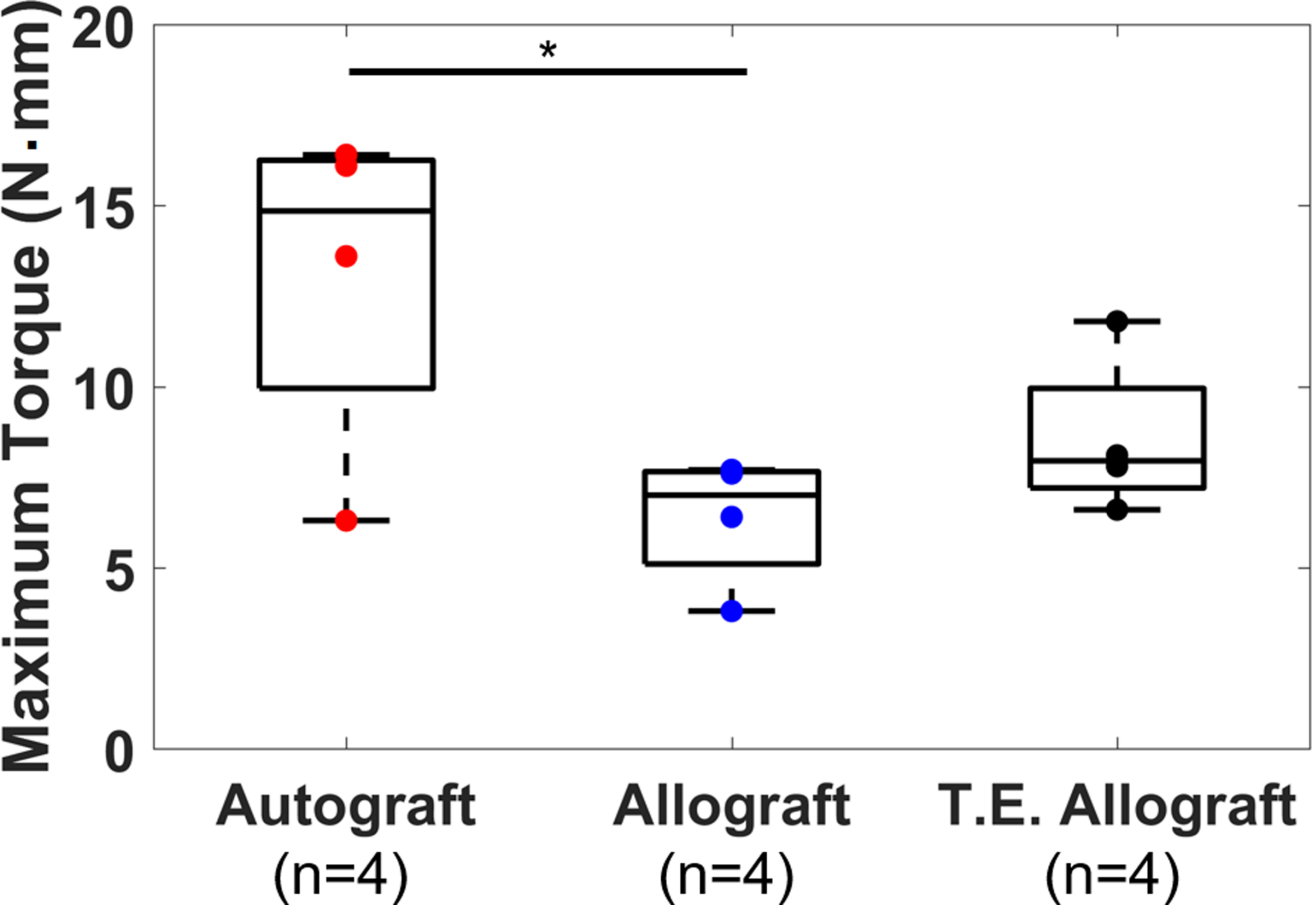
Maximum torque measured by biomechanical testing. * indicates that the difference between autograft group and allograft group is statistically significant.

Biomechanical testing results are shown in Fig 3. The autograft group showed the highest average maximum torque (13.1 ± 4.7 N·m, mean ± standard deviation) versus the allograft (6.3 ± 1.8 N·m) and T.E. allograft (8.6 ± 2.2 N·m) groups. One-way ANOVA followed by Bonferroni correction showed differences between the autograft and allograft groups are statistically significant (*p* = 0.015). This trend agrees with previous studies: the maximum torques were significantly different between the autograft and allograft group, but not between the T.E. allograft and allograft groups [6, 8]. In this study, however, the average maximum torque of the autograft group is smaller than the maximum torque observed previously [8]. This discrepancy may be due to the difference in strains of host mouse (BALB/c in this study versus C57BL/6 in previous studies). Note that the maximum torque of one mouse from the autograft group is unusually low compared to the rest of autografts.

### Spatial distribution of blood flow changes

**Fig 4:**
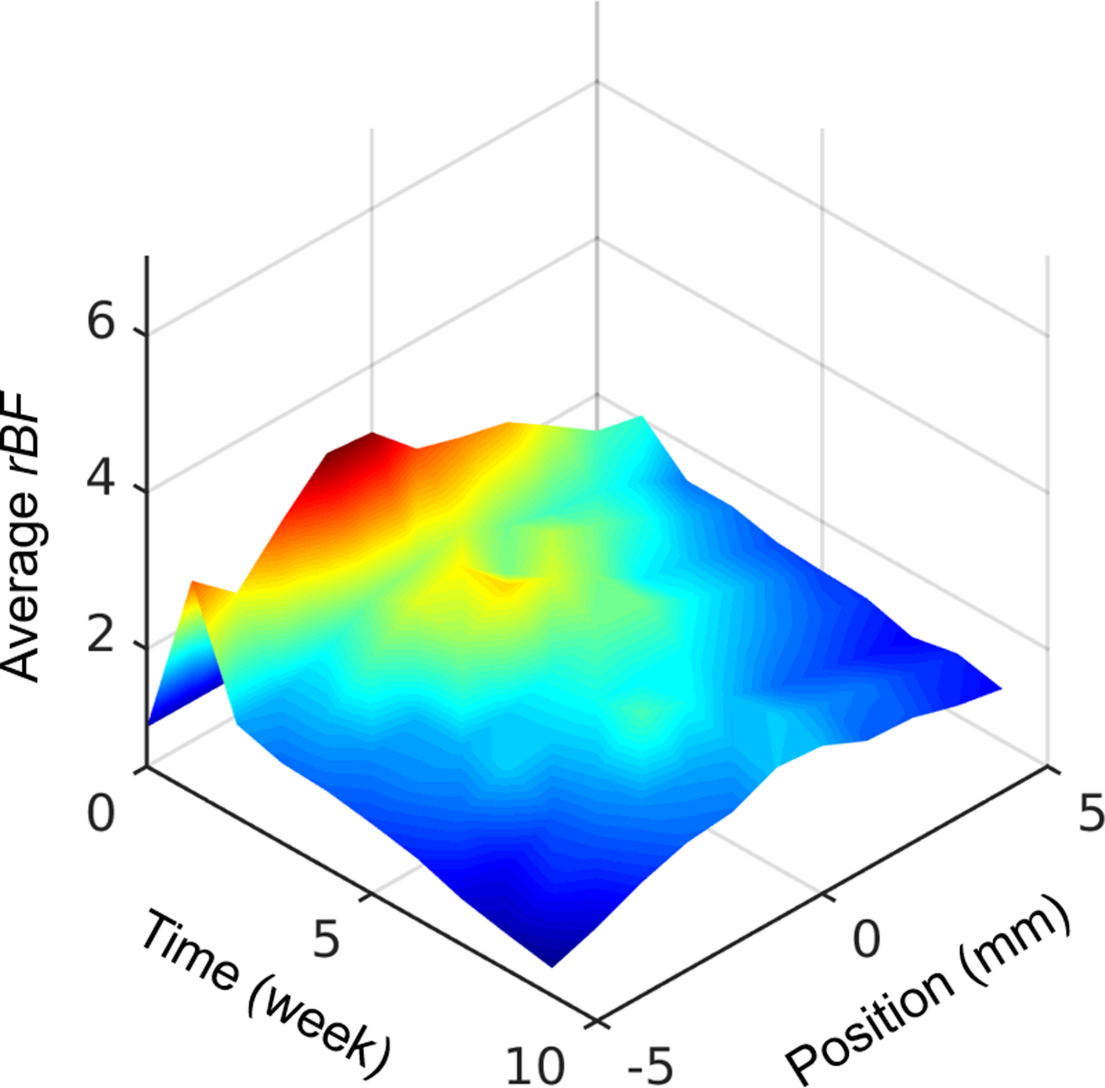
Blood flow changes in different femur segments over a 9-week healing. Average *rBF* was calculated as the average of all mice (N = 19). Position on the femur is illustrated in Fig 2, with the center of the graft as the origin.

The temporal changes of *rBF*(*l*,*t*;*i*) along the femur are shown in Fig 4 as a surface plot. Fig 4 shows average *rBF* of all mice from week 0 to 9 and femur position -5 to 5 mm. As can be seen in Fig 2, the graft region is located between -2 and 2 mm. Fig 4 shows the blood flow changes were mainly localized to the graft region on average, with some notable early changes in the proximal end. In this study, we focus on the blood flow changes in the graft region which is the region of interest.

### Temporal blood flow trends

To investigate the temporal trends in blood flow changes, we concentrated on temporal changes of the average *rBF* in the graft region (-2 ≤ *l* ≤ 2), as it is the region where major changes were observed. The graft *rBF* (*rBF*^*g*^) from all 19 mice were utilized in the following analysis.

To examine whether the blood flow at different weeks were related, the correlation coefficients of *rBF*^g^ at different weeks were calculated. Before calculating the correlation coefficient, the Shapiro-Wilk normality test was performed. The test indicated that the distributions were skewed and significantly different from a normal distribution (S2 Table). Logarithm transform was performed to alleviate the skewness of the data, yielding log(*rBF*^g^) (S3 Table), as verified by Shapiro-Wilk normality test.

The pair-wise Pearson correlation coefficients (*r*) of the log(*rBF*^*g*^) at different weeks are listed in Table 1. The results in Table 1 show that the log(*rBF*^*g*^) from the different weeks are correlated (*r* > 0.4). After week 5, log(*rBF*^*g*^) are highly correlated with each other (*r* > 0.7), which indicated the blood flow changes after week 5 follow similar temporal trends. The lower correlation in the first 5 weeks indicates the differences in temporal trends among the mice are larger in the early weeks after intervention. Altogether the data indicate that blood flow changes measured in the first 5 weeks is sufficient to represent overall temporal trends.

**Table 1:** Correlation coefficients of log(*rBF*^g^) at different weeks of all mice (N = 19). (**Bold**: correlation coefficient > 0.7).

Log(rBF^g^)	Week								
	1	2	3	4	5	6	7	8	9
Week 1	**1.00**	—	—	—	—	—	—	—	—
Week 2	0.41	**1.00**	—	—	—	—	—	—	—
Week 3	0.61	**0.77**	**1.00**	—	—	—	—	—	—
Week 4	0.45	**0.76**	0.67	**1.00**	—	—	—	—	—
Week 5	0.46	0.64	0.60	**0.81**	**1.00**	—	—	—	—
Week 6	0.51	0.49	0.51	**0.71**	**0.87**	**1.00**	—	—	—
Week 7	0.68	0.61	0.66	**0.81**	**0.78**	**0.75**	**1.00**	—	—
Week 8	0.53	0.50	0.49	0.66	**0.80**	**0.83**	**0.87**	**1.00**	—
Week 9	0.46	0.53	0.42	**0.79**	**0.79**	**0.78**	**0.82**	**0.84**	**1.00**

### Individual and group-averaged blood flow trends of different graft groups

**Fig 5:**
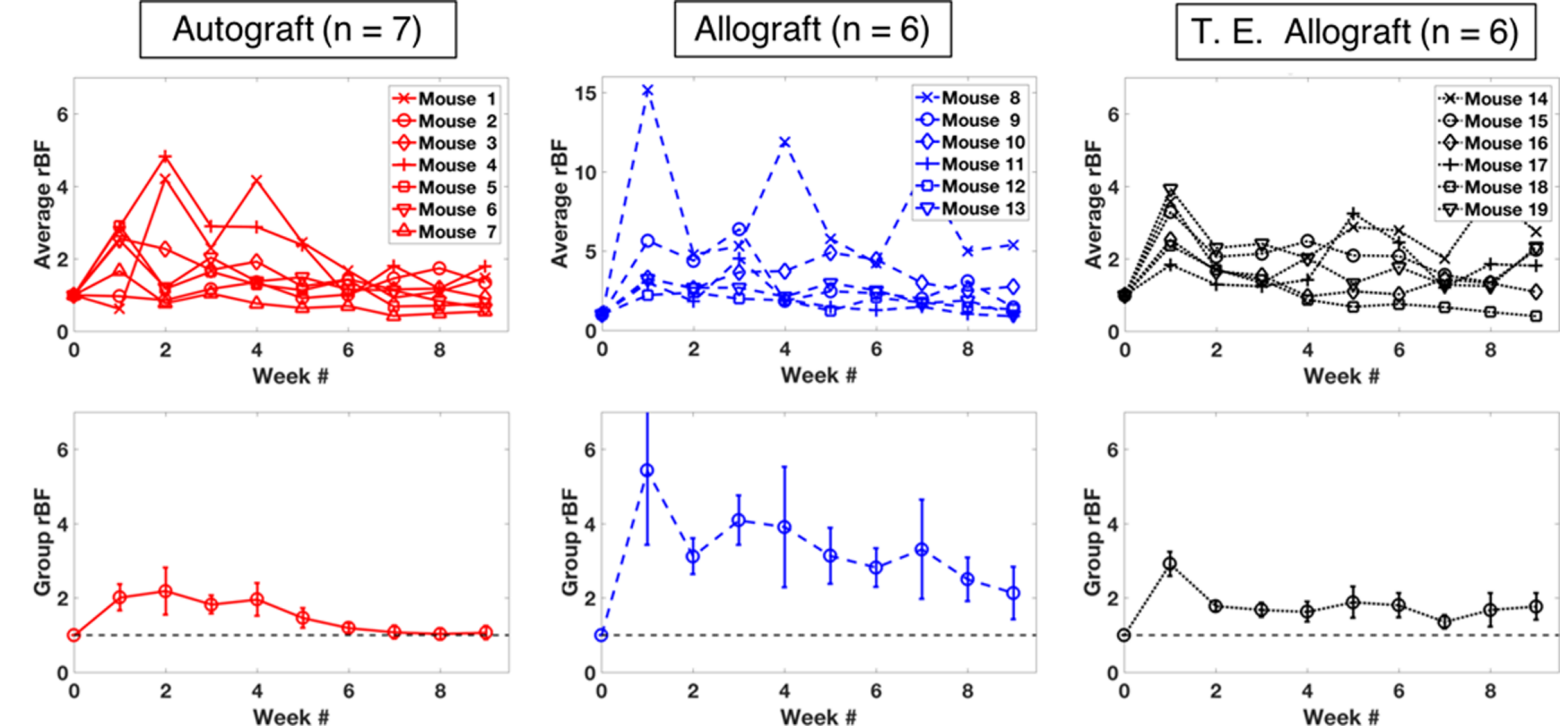
Average blood flow in the graft region (*rBF*^g^) during the 9 weeks of healing. The first row shows the individual temporal changes in the different graft groups, and the second row shows the corresponding group average with the standard error of the mean. In order to show the full range of temporal changes for the individual mice in the allograft group, a different *rBF* range is chosen (top middle plot), whereas other plots are fixed with the same *rBF* range.

The temporal changes in *rBF*^g^ for each mouse are shown in the first row in Fig 5, grouped by graft type. Group-averaged changes are shown in the second row. The individual blood flow changes in the first row show that, in the autograft group, *rBF*^*g*^ increased rapidly by week 1 or 2 after the graft transplantation followed by a gradual decrease over time. By week 6, the average *rBF* reached baseline levels. In the allograft group, the time to reach the maximum *rBF* ranged from 1 to 5 weeks, showing greater variability compared to autografts. The maximum blood flow elevation was also larger in some of the allografts compared to the autografts. In the T.E. allograft group, most mice showed *rBF*^g^ peaks at week 1 except mouse 14 and 17, which showed higher peaks at week 5.

These trends are in line with our previous experiment [17], except that current autografts exhibit more variabilities in temporal profile. This may be due to variable surgical skills. Indeed, one of the autografts had a maximum torque similar to that of allografts. These variabilities can be taken into account if the success of individual graft integration is assessed by biomechanical testing instead of graft type.

### Temporal blood flow change grouped by healing outcome

**Fig 6:**
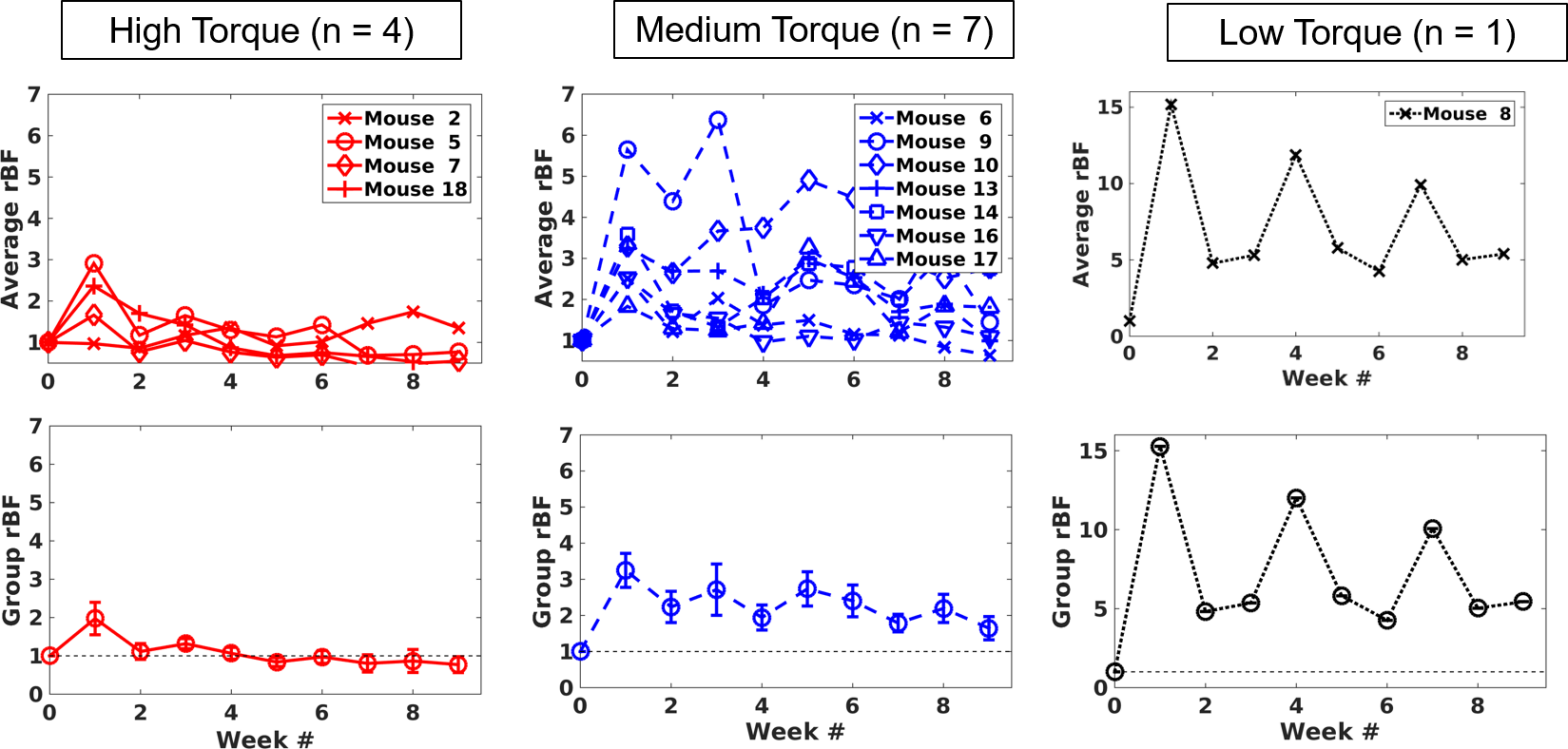
Average blood flow in the graft region (*rBF*^g^) during the 9 weeks of healing based on the measured maximum torque. The range of maximum torque for the high, medium, and low torque groups are >10, 5–10, and < 5 N·mm, respectively. The first row shows individual changes and the second row shows corresponding group averages. Note the y range for the low torque group is much larger than the others.

To show how the blood flow changes may be related to biomechanical testing results, biomechanics data were regrouped into three groups. The high torque group included mice with maximum torque larger than 10 N·mm (Mouse 2, 5, 7 from the autograft group and Mouse 18 from the T.E. allograft group), the medium torque group include mice with maximum torque between 5–10 N·mm (Mouse 6 from the autograft group, Mouse 9, 10, 13 from the allograft group, and Mouse 14, 17, 18 from the T.E. allograft group), and the low torque group with maximum torque smaller than 5 N·mm (Mouse 8 from the allograft group). The temporal changes for each mouse are plotted in the first row of Fig 6, while the group-averaged results are shown in the second row. The results show very clear trends; for example, the high torque group exhibits more modest blood flow elevations compared to other groups. Note that the plots for the low torque group have much larger *rBF* ranges (y axis) than the other groups to show the data in its entirety.

### Healing prediction model using partial least squares (PLS) regression

Analysis of temporal changes in blood flow shows that dramatic changes mainly occurred over five weeks of healing. With this insight, a predictive model was constructed using only early blood flow changes (i.e., 1–5 weeks). Using the blood flow information in the first 5 weeks also has the implication of potential for early prediction of bone healing, as the first 5 weeks enables much earlier evaluation of healing diagnosis as compared to 9 weeks.

**Fig 7:**
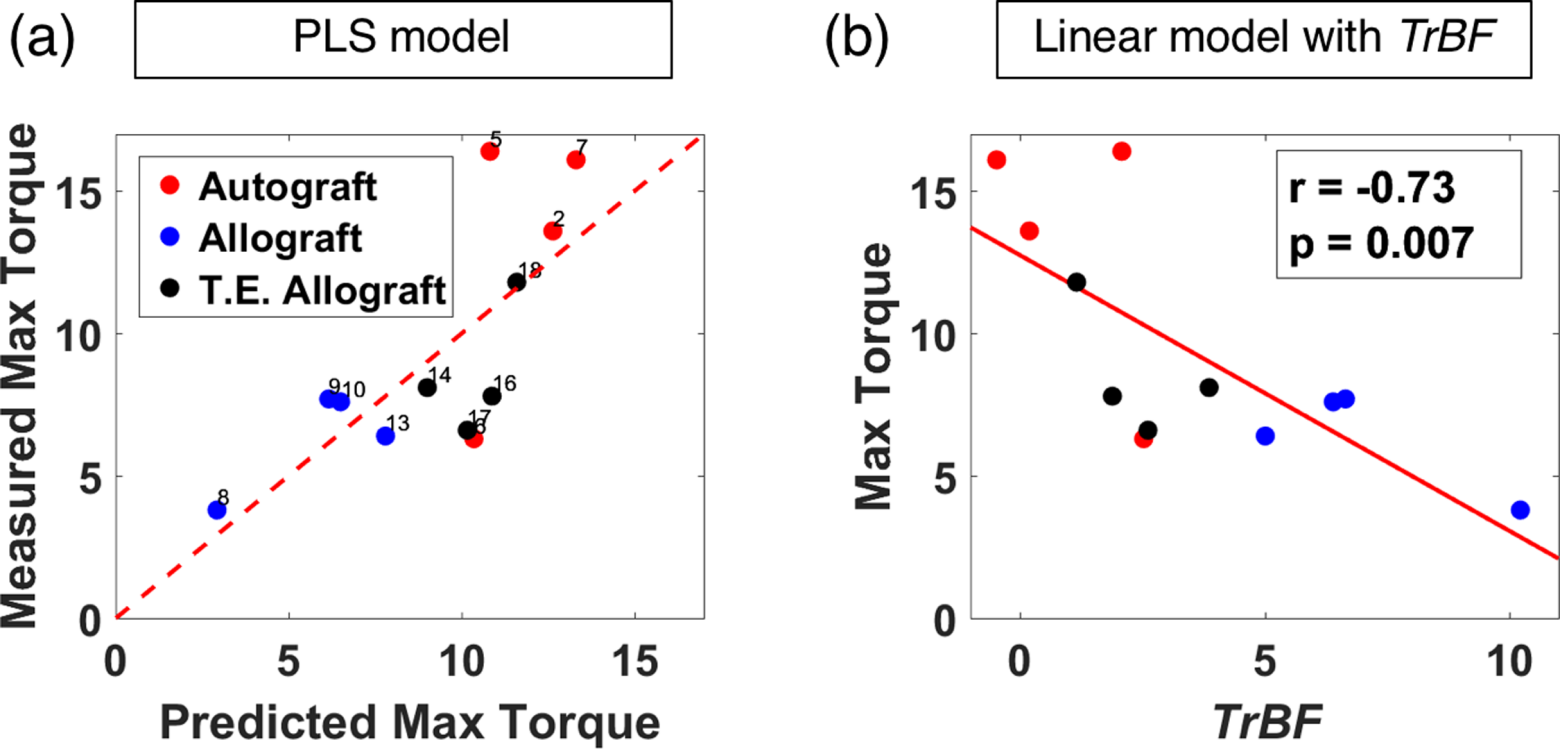
Prediction results on bone maximum torque. (a) Partial Least Squares (PLS) predicted maximum torque versus measured results. Data from the autograft, allograft, and T.E. allograft group are marked with red, blue and black, respectively. The dashed lines are the 1:1 line. (b) Linear correlation between *TrBF* and measure maximum torque.

PLS regression found the optimal multivariate model with linear combinations of *rBF*^g^ from week 1 to week 5 using the criterion of minimum mean PRESS, as described in the Methods section. The VIP values for different log(*rBF*^g^) ranged from 0.87 to 1.2, indicating they all have important contributions to this PLS model. The resulting prediction formula is:

$$y=12.75-0.88x_{1}-1.20x_{2}-0.95x_{3}-0.85x_{4}-1.04x_{5},$$ (2)

where *y* is the predicted maximum torque, *x*_*t*_ is log(*rBF*^g^) at week *t* with *t* ranging from 1 to 5. A plot of the predicted maximum torque and measured results are shown in Fig 7A. The result shows good correspondence between predicted and measured maximum torque.

In the prediction formula generated from PLS regression, the coefficients of log(*rBF*^g^) ranged from -0.8 to -1.2, which indicated the log(*rBF*^g^) data at each week were inversely proportional to the maximum torque. More importantly, the absolute values of the coefficients were close to 1, behaving like a sum of log(*rBF*^g^). With this insight, a total blood flow change, *TrBF*^*g*^ was defined, as follows,

$$TrBF^{g}=\sum_{t=1}^{5}\log\left(rBF^{g}\left(t\right)\right).$$ (3)

*TrBF*^*g*^ is a summation of log(*rBF*) from week 1 to week 5. It quantifies the total increase from baseline in blood flow measured by DCT in the graft region (note log(x) ≈ x-1 at x = 1). The correlation between *TrBF* and maximum torque is shown in Fig 7B. The Pearson’s correlation coefficient is -0.73 (p = 0.007), which clearly indicates an inverse correlation between healing outcome and total blood flow increase observed by DCT.

## Discussion

In this study, a predictive model for femoral graft healing was developed utilizing longitudinal blood flow changes monitored via DCT. DCT noninvasively tracked weekly blood flow changes in the mouse leg prior to graft transplantation and through 9 weeks post-transplantation. Healing was evaluated biomechanically at 9 weeks after graft transplantation. With this study, the potential to use blood flow-derived parameters to predict long-term healing outcomes was demonstrated with the inverse correlation between maximum torque after 9 weeks of healing and *TrBF*. In addition, the spatial and temporal blood flow changes were characterized, revealing major blood flow changes occur in the graft region during the first 5 weeks after graft transplantation.

The use of the prediction model led to the revelation of an inverse correlation between maximum torque and total blood flow elevation: if there is greater cumulative blood flow elevation throughout the first 5 weeks of healing, the maximum torque is lower at week 9. The temporal trends of *rBF* sorted by maximum torque also support this inverse correlation, as shown in Fig 6. The inflammatory stage of the bone injury healing process may explain the inverse correlation between the measured blood flow and biomechanical parameters. It is well established that acute inflammation is critical for bone healing [32]. Dysregulated or chronic inflammation, however, has been shown to disrupt bone repair and remodeling, which results in diminished bone strength [32]. The elevated blood flow observed in the present study is presumably attributed to inflammation; therefore, bone weakness associated with increased blood flow over time may be due to impaired restoration of mechanics. Thus, prolonged blood flow elevation may serve as an indicator of poor bone healing.

The correlation between predicted maximum torque and measured results highlights the ability of our method to make individual diagnoses. The spatial and temporal trends in *rBF* confirm that blood flow changes mainly occur in the early stage of healing, strongly supporting the potential for early prediction with DCT.

Despite the new findings, there are also several limitations. In the current study, weekly measurements were performed and results show blood flow changes among groups in the first five weeks after the graft transplantation. Our main observation in temporal trends is a blood flow elevation and a subsequent decrease. However, in some mice, *rBF* changes with more than one peak in average blood flow were observed (Fig 5), especially in the allograft group. In the future, more frequent temporal measurements may help to reveal more detailed temporal features in blood flow changes. In addition, for the current study, blood flow changes in tissue juxtaposed to the graft were utilized to build the prediction model. Our DCT results also show that there are changes in blood flow at the proximal and distal tissues, and the autograft group and allograft group have different spatial patterns. The importance of blood flow spatial distribution in soft tissue remains to be investigated.

Additionally, to quantify the healing outcomes, maximum torque measurements from biomechanical testing were used. The biomechanical testing suffered from sample attrition during preparation (e.g., removal of intramedullary pins). Therefore, the prediction model was developed based on 12 samples in the current study. Optimizing sample preparation for biomechanical testing will minimize sample attrition in the future. No gross damage was observed in the tested samples. However, to minimize the possibility of minor damage during sample preparation, steps will be taken to evaluate the integrity of the bone after intramedullary pin removal. Although maximum torque had been proven to be a valuable and repeatable metric, it does not provide the complete evaluation of healing [9]. In the future, a complete assessment of healing may be beneficial by using high-resolution micro-CT on isolated femur samples to quantify callus volume and nonunion ratio, in addition to biomechanical testing [9]. For the prediction model development, a more conservative VIP threshold value (e.g., 1.0) [33] could be utilized to further reduce the number of predictors to avoid overfitting. With these improvements, a more robust prediction model will be developed and tested using more samples.

As a new technique for blood flow imaging, DCT is advantageous in its non-invasiveness and low cost. However, we acknowledge there are technical complications in the DCT measurements that need to be addressed, as detailed in our previous publication [34]. Despite these limitations, the current research demonstrates that DCT-measured blood flow changes after graft transplantation can be utilized to predict long-term healing outcomes in a mouse femoral graft model, showing its potential to help accelerate tissue engineering-based interventions. Furthermore, the methodologies developed in this study including instrumentation, image reconstruction algorithm and development of the prediction model can be modified and extended for monitoring human bone healing. Note that the current DCT setup with a minor modification can readily be utilized to monitor bone healing processes in human subjects for bone grafts or fractures at shallow depths (2–3 mm).

## Conclusion

Blood flow changes measured using a noninvasive DCT system were utilized to predict healing outcomes of mouse femoral grafts. Temporal differences in graft blood flow changes among mice mainly occur during the first five weeks after graft transplantation. Prediction models built using partial least squares regression reveal that lower bone strength in the healed femur is correlated with temporally extended blood flow elevation in the femoral graft during the healing process, which may be a sign of poor healing. The model demonstrates that blood flow can be utilized to extract useful information to predict graft healing.

## Supporting information

S1 Table Longitudinal average rBF for each 1mm-long femur segment of individual mice. (XLSX)

S2 Table Longitudinal rBF^g^ of individual mice. (DOCX)

S3 Table Longitudinal log(rBF^g^) of individual mice. (DOCX)

S4 Table The ARRIVE guidelines checklist. (PDF)

S1 Fig Data analysis workflow and data used in the different analyses. (DOCX)
