# Supplementary material for: Temporal blood flow changes measured by diffuse correlation tomography predict murine femoral graft healing
Source: PLoS One. 2018 May 29;13(5):e0197031. doi: 10.1371/journal.pone.0197031 (PMC5973582; doi:10.1371/journal.pone.0197031)
Supplement: S2 Table — (DOCX) [file pone.0197031.s002.docx]

**S2 Table**. **Longitudinal *rBF*^g^ of individual mice.** (NA: not available)

| Graft Type | # | Week | | | | | | | | | | Max Torque (N·mm) |
| --- | --- | --- | --- | --- | --- | --- | --- | --- | --- | --- | --- | --- |
|  |  | 0 | 1 | 2 | 3 | 4 | 5 | 6 | 7 | 8 | 9 |  |
| Autograft | 1 | 1.00 | 0.61 | 4.24 | 2.31 | 4.22 | 2.45 | 1.66 | 0.91 | 1.09 | 1.47 | NA |
|  | 2 | 1.00 | 0.97 | 0.85 | 1.18 | 1.37 | 0.92 | 1.03 | 1.45 | 1.75 | 1.36 | 13.6 |
|  | 3 | 1.00 | 2.58 | 2.27 | 1.70 | 1.93 | 1.24 | 1.44 | 1.16 | 1.16 | 0.93 | NA |
|  | 4 | 1.00 | 2.91 | 4.91 | 2.92 | 2.91 | 2.39 | 0.97 | 1.82 | 1.18 | 1.79 | NA |
|  | 5 | 1.00 | 2.91 | 1.16 | 1.64 | 1.29 | 1.13 | 1.42 | 0.68 | 0.70 | 0.77 | 16.4 |
|  | 6 | 1.00 | 2.50 | 1.21 | 2.04 | 1.37 | 1.49 | 1.15 | 1.12 | 0.83 | 0.63 | 6.3 |
|  | 7 | 1.00 | 1.65 | 0.76 | 1.04 | 0.75 | 0.63 | 0.69 | 0.42 | 0.48 | 0.54 | 16.1 |
| Allograft | 8 | 1.00 | 15.28 | 4.82 | 5.36 | 12.01 | 5.81 | 4.26 | 10.07 | 5.03 | 5.44 | 3.8 |
|  | 9 | 1.00 | 5.73 | 4.46 | 6.49 | 1.87 | 2.49 | 2.36 | 2.02 | 3.16 | 1.45 | 7.7 |
|  | 10 | 1.00 | 3.30 | 2.64 | 3.69 | 3.79 | 4.94 | 4.53 | 3.01 | 2.52 | 2.78 | 7.6 |
|  | 11 | 1.00 | 2.97 | 1.82 | 4.48 | 1.96 | 1.47 | 1.26 | 1.49 | 1.02 | 0.89 | NA |
|  | 12 | 1.00 | 2.22 | 2.38 | 1.99 | 1.88 | 1.22 | 2.06 | 1.76 | 1.48 | 1.32 | NA |
|  | 13 | 1.00 | 3.26 | 2.69 | 2.69 | 2.13 | 2.99 | 2.53 | 1.71 | 1.89 | 0.99 | 6.4 |
| T.E. Allograft | 14 | 1.00 | 3.57 | 1.70 | 1.33 | 2.05 | 2.90 | 2.79 | 2.00 | 3.78 | 2.75 | 8.1 |
|  | 15 | 1.00 | 3.28 | 2.06 | 2.15 | 2.49 | 2.09 | 2.08 | 1.56 | 1.35 | 2.25 | NA |
|  | 16 | 1.00 | 2.53 | 1.63 | 1.51 | 0.96 | 1.11 | 1.03 | 1.44 | 1.32 | 1.08 | 7.8 |
|  | 17 | 1.00 | 1.84 | 1.32 | 1.24 | 1.41 | 3.23 | 2.43 | 1.21 | 1.86 | 1.84 | 6.6 |
|  | 18 | 1.00 | 2.36 | 1.68 | 1.41 | 0.86 | 0.67 | 0.75 | 0.66 | 0.52 | 0.41 | 11.8 |
|  | 19 | 1.00 | 3.96 | 2.29 | 2.42 | 2.03 | 1.33 | 1.78 | 1.29 | 1.25 | 2.35 | NA |
| Shapiro-Wilk test | | NA | <0.001 | 0.016 | 0.003 | <0.001 | 0.009 | 0.016 | <0.001 | 0.003 | 0.002 | 0.071 |
