# Supplementary material for: Temporal blood flow changes measured by diffuse correlation tomography predict murine femoral graft healing
Source: PLoS One. 2018 May 29;13(5):e0197031. doi: 10.1371/journal.pone.0197031 (PMC5973582; doi:10.1371/journal.pone.0197031)
Supplement: S3 Table — (DOCX) [file pone.0197031.s003.docx]

**S3 Table**. **Longitudinal log(*rBF*^g^) of individual mice.** (NA: not available)

| Graft  Type | # | Week | | | | | | | | | Maximum Torque (N·mm) |
| --- | --- | --- | --- | --- | --- | --- | --- | --- | --- | --- | --- |
|  |  | 1 | 2 | 3 | 4 | 5 | 6 | 7 | 8 | 9 |  |
| Autograft | 1 | -0.50 | 1.45 | 0.84 | 1.44 | 0.90 | 0.51 | -0.09 | 0.09 | 0.38 | NA |
|  | 2 | -0.03 | -0.16 | 0.16 | 0.31 | -0.09 | 0.02 | 0.37 | 0.56 | 0.30 | 13.6 |
|  | 3 | 0.95 | 0.82 | 0.53 | 0.66 | 0.22 | 0.36 | 0.14 | 0.15 | -0.08 | NA |
|  | 4 | 1.07 | 1.59 | 1.07 | 1.07 | 0.87 | -0.03 | 0.60 | 0.17 | 0.58 | NA |
|  | 5 | 1.07 | 0.15 | 0.49 | 0.26 | 0.12 | 0.35 | -0.38 | -0.35 | -0.27 | 16.4 |
|  | 6 | 0.92 | 0.19 | 0.71 | 0.32 | 0.40 | 0.14 | 0.11 | -0.19 | -0.46 | 6.3 |
|  | 7 | 0.50 | -0.27 | 0.04 | -0.29 | -0.46 | -0.36 | -0.88 | -0.73 | -0.61 | 16.1 |
| Allograft | 8 | 2.73 | 1.57 | 1.68 | 2.49 | 1.76 | 1.45 | 2.31 | 1.61 | 1.69 | 3.8 |
|  | 9 | 1.75 | 1.49 | 1.87 | 0.63 | 0.91 | 0.86 | 0.70 | 1.15 | 0.37 | 7.7 |
|  | 10 | 1.20 | 0.97 | 1.31 | 1.33 | 1.60 | 1.51 | 1.10 | 0.93 | 1.02 | 7.6 |
|  | 11 | 1.09 | 0.60 | 1.50 | 0.67 | 0.39 | 0.23 | 0.40 | 0.02 | -0.11 | NA |
|  | 12 | 0.80 | 0.87 | 0.69 | 0.63 | 0.20 | 0.72 | 0.56 | 0.39 | 0.27 | NA |
|  | 13 | 1.18 | 0.99 | 0.99 | 0.76 | 1.10 | 0.93 | 0.54 | 0.64 | -0.01 | 6.4 |
| T.E.  Allograft | 14 | 1.27 | 0.53 | 0.29 | 0.72 | 1.06 | 1.03 | 0.69 | 1.33 | 1.01 | 8.1 |
|  | 15 | 1.19 | 0.72 | 0.77 | 0.91 | 0.74 | 0.73 | 0.44 | 0.30 | 0.81 | NA |
|  | 16 | 0.93 | 0.49 | 0.41 | -0.04 | 0.10 | 0.03 | 0.36 | 0.28 | 0.08 | 7.8 |
|  | 17 | 0.61 | 0.28 | 0.22 | 0.35 | 1.17 | 0.89 | 0.19 | 0.62 | 0.61 | 6.6 |
|  | 18 | 0.86 | 0.52 | 0.35 | -0.16 | -0.40 | -0.29 | -0.42 | -0.65 | -0.88 | 11.8 |
|  | 19 | 1.38 | 0.83 | 0.88 | 0.71 | 0.28 | 0.58 | 0.26 | 0.23 | 0.85 | NA |
| Shapiro-Wilk test | | 0.056 | 0.527 | 0.390 | 0.083 | 0.735 | 0.802 | 0.047 | 0.875 | 0.998 | 0.071 |
