# Supplementary figures and images for: Temporal blood flow changes measured by diffuse correlation tomography predict murine femoral graft healing
Source: PLoS One. 2018 May 29;13(5):e0197031. doi: 10.1371/journal.pone.0197031 (PMC5973582; doi:10.1371/journal.pone.0197031)

**
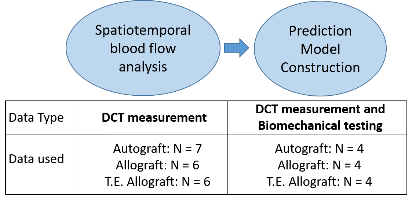
**

**S4 Fig. Data analysis workflow and data used in the different analyses.**

Supplement: S1 Fig — (DOCX) [file pone.0197031.s005.docx]
